# Sound the (Smaller) Alarm: The Triphosphate Magic Spot Nucleotide pGpp

**DOI:** 10.1128/iai.00432-22

**Published:** 2023-03-15

**Authors:** Areej Malik, Megan A. Hept, Erin B. Purcell

**Affiliations:** a Old Dominion University, Department of Chemistry and Biochemistry, Norfolk, Virginia, USA; University of Pittsburgh

**Keywords:** (p)ppGpp, alarmone, bacterial nucleotide signaling, pGpp, stringent response

## Abstract

It has recently become evident that the bacterial stringent response is regulated by a triphosphate alarmone (pGpp) as well as the canonical tetra- and pentaphosphate alarmones ppGpp and pppGpp [together, (p)ppGpp]. Often dismissed in the past as an artifact or degradation product, pGpp has been confirmed as a deliberate endpoint of multiple synthetic pathways utilizing GMP, (p)ppGpp, or GDP/GTP as precursors. Some early studies concluded that pGpp functionally mimics (p)ppGpp and that its biological role is to make alarmone metabolism less dependent on the guanine energy charge of the cell by allowing GMP-dependent synthesis to continue when GDP/GTP has been depleted. However, recent reports that pGpp binds unique potential protein receptors and is the only alarmone synthesized by the intestinal pathogen Clostridioides difficile indicate that pGpp is more than a stand-in for the longer alarmones and plays a distinct biological role beyond its functional overlap (p)ppGpp.

## INTRODUCTION

**FIG 1:**
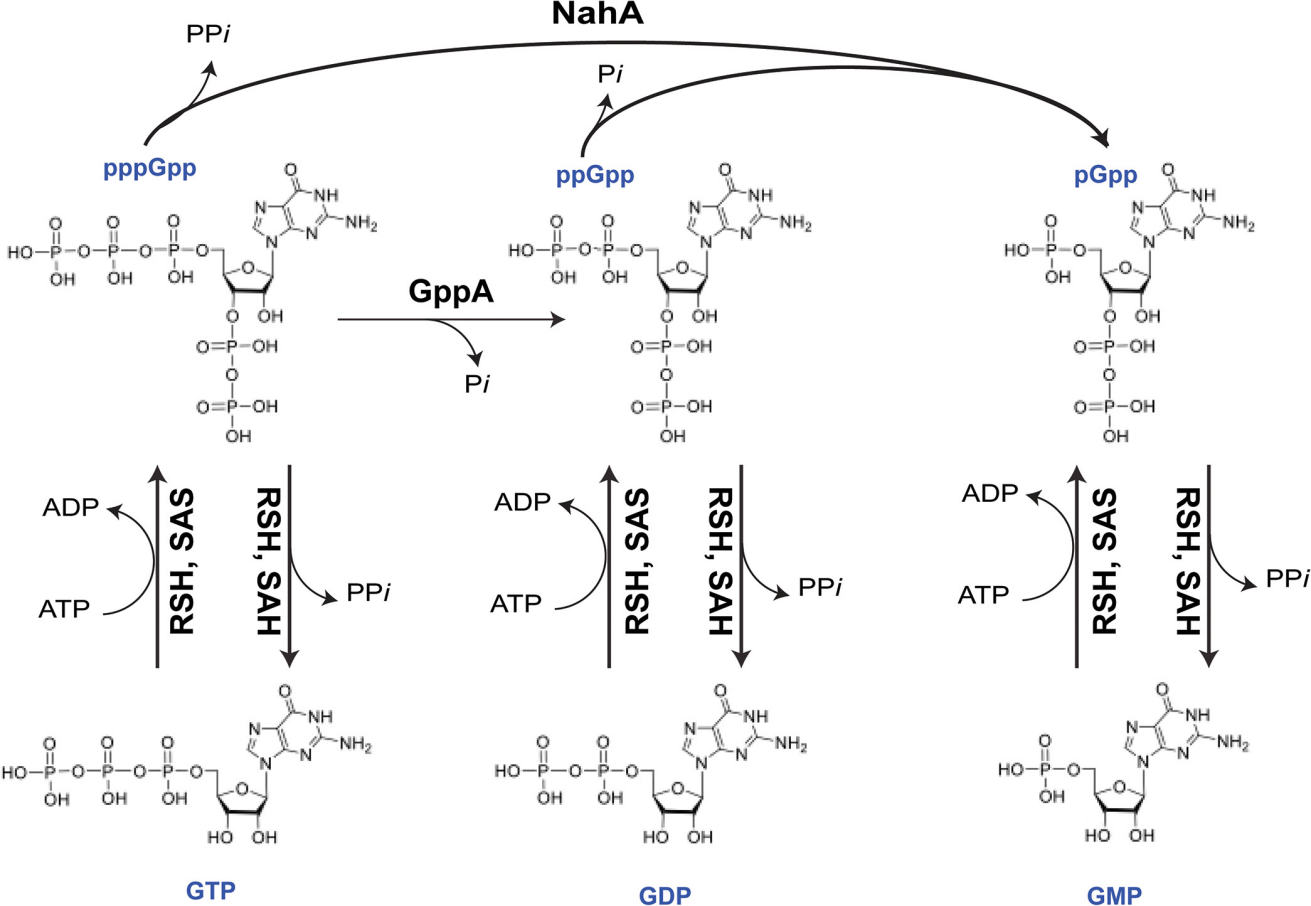
Structures and synthesis of guanosine alarmones. Shown are the three guanoine alarmones, pppGpp, ppGpp, and pGpp. Alarmone synthesis from GTP/GDP/GMP or (p)ppGpp precursors is catalyzed by synthetase enzymes and alarmone hydrolysis, and modification is catalyzed by hydrolase enzymes. Direct synthesis of pGpp from GTP/GDP in Clostridium difficile is omitted for clarity.

Nucleotide alarmones, whose ribose sugar moieties are phosphorylated at two sites (Fig. 1), regulate bacterial gene expression and cellular processes. Low alarmone concentrations enforce metabolic homeostasis in some species, ensuring balanced outputs by related synthetic pathways (1 to 8). Almost all bacterial species are capable of mounting the stringent response. The few species that are not are mainly obligate intracellular pathogens or endosymbionts that colonize very stable environments (9, 10). During the stringent response, a detrimental stimulus such as stationary-phase onset, nutrient starvation, cell-envelope stress, or exposure to antibiotics triggers the rapid synthesis and accumulation of guanosine alarmones (11 to 14). These interact with protein or RNA effectors to halt “housekeeping” transcriptional activity and cell replication and redirect the cell’s resources to crisis response mechanisms. The specific stimuli that provoke alarmone synthesis vary widely based on the environmental niches and metabolic needs of diverse bacteria (7, 10, 15 to 21). Similarly, the effectors that respond to alarmone accumulation can differ greatly between clades and species (5, 22). It has recently been determined that many bacteria maintain a low but nonzero concentration of alarmones during unstressed growth in nutrient-rich media, and that small and/or gradual increases in basal alarmone levels can have a profound regulatory effect on metabolism and cell growth (6 to 8, 23). Some alarmone-regulated genes respond to fluctuations in basal levels while others are activated only by the dramatic escalations necessary to induce the stringent response (7, 8, 24).

It was originally thought that the stringent response was mediated by two closely related molecules, the “magic spot” nucleotides whose profound effect on cellular behavior was apparent well before their chemical structures had been defined (25). These molecules were found to be present on autoradiograms of separated bacterial cytoplasm only when cells dosed with radioactive phosphate were then starved, and were described as even more “sluggish” on chromatograms than GTP (25 to 28) and were soon identified as guanosine 3′-diphosphate 5′-diphosphate (guanosine tetraphosphate or ppGpp) and guanosine 3′-diphosphate 5′-triphosphate (guanosine pentaphosphate or pppGpp) (Fig. 1) (25, 26, 28). Together, these canonical alarmones are known as (p)ppGpp. ppGpp and pppGpp largely regulate the same processes with a high degree of functional redundancy, although some organisms respond more strongly to one form and some alarmone-binding protein effectors are specific for the tetraphosphate or pentaphosphate forms (29 to 33).

Alarmones are generated by synthetase enzymes that transfer a pyrophosphate from an ATP to the 3′-hydroxyl group of a GDP or GTP substrate (Fig. 1). Most synthetase enzymes will utilize either GDP or GTP as the substrate but have a higher affinity for one of the nucleotides (13, 34 to 40). As the ratio of GDP to GTP in bacterial cytoplasm is a dynamic parameter that changes quickly in response to nutrient conditions, the relative production of ppGpp and pppGpp could reflect substrate availability when alarmone synthesis is activated (26, 29).

Hyperphosphorylated purine molecules beyond (p)ppGpp have been recognized for some time (41). Some synthetases are capable of utilizing ATP as a phosphoacceptor as well as a phosphodonor to generate adenosine 3′-diphosphate 5′-triphosphate (adenosine pentaphosphate or pppApp) (42, 43). A triphosphate guanosine alarmone, guanosine 3′-disphosphate 5′-monophosphate (pGpp), was difficult to detect on early chromatograms as it migrates very closely to GTP, but a third “magic spot” was identified in the cytoplasm of nutrient-limited Bacillus subtilis in 1979 (27, 44). Pyrophosphotransfer to mono-, di-, and triphosphate adenosine and guanosine molecules has been documented for decades, but only (p)ppGpp was consistently detected *in vivo* and conclusively linked to a phenotype, and the small amounts of pGpp detected in some assays were easy to dismiss as artifacts or unimportant (41). It has recently been confirmed that some synthetase enzymes are capable of utilizing GMP as a substrate to synthesize pGpp directly (13, 33, 37, 39, 40, 45 to 47). In addition, hydrolase enzymes can selectively remove 5′-phosphates from longer guanosine alarmones (29, 33, 43, 48 to 50).

It was initially suggested that the use of GMP as a substrate would enable alarmone synthesis to continue when cytoplasmic GDP and GTP have been depleted (38, 51, 52). This view positions pGpp as an understudy for the longer canonical alarmones, perhaps less potent but capable of performing the same role. Indeed, pGpp appears to bind many alarmone effectors with lower affinity than (p)ppGpp and to influence multiple processes also regulated by the longer alarmones. (31 to 33, 53). However, evidence quickly emerged that pGpp is an independently active regulator rather than a merely tolerable substitute for (p)ppGpp. Synthetases that generate pGpp preferentially or exclusively have been characterized, and putative effectors that bind pGpp preferentially or exclusively have been identified in pulldown assays (31, 39, 40).

There have been several excellent recent reviews of the bacterial stringent response, with comprehensive coverage of the genetic regulons influenced by alarmone levels and the currently characterized alarmone synthetases, hydrolases, and protein effectors (20, 30, 54 to 56). Some explicitly limit their scope to (p)ppGpp, while others acknowledge the existence of and relative dearth of information about pGpp. However, it has become clear that the treatment of pGpp as an adjunct alarmone that exists mainly or only to amplify the signals produced by (p)ppGpp is incomplete. This review will summarize the current knowledge of pGpp metabolism and functionality, with a focus on the evidence that pGpp has functions distinct from those of (p)ppGpp and merits consideration as an individual signaling molecule as well as a generic (pp)pGpp alarmone.

## ALARMONE METABOLISM AND SENSING

### Bifunctional Rel-Spo hydrolases.

In Escherichia coli and other Gram-negative bacteria, alarmone synthesis is mediated by the (p)ppGpp synthetase RelA and the bifunctional synthetase/hydrolase SpoT, closely related multidomain proteins whose C termini contain noncatalytic regulatory domains (10). Gram-positive bacteria typically encode a single bifunctional alarmone synthetase-hydrolase with the same conserved domains as RelA and SpoT, known either as Rel or as the Rel-Spo homolog (RSH) (35, 57).

Synthetase enzymes catalyze the magnesium-dependent transfer of a pyrophosphate from an ATP substrate to the 3’OH of a guanosine nucleotide precursor (58, 59). GDP is converted to a guanosine tetraphosphate alarmone, while GTP becomes a pentaphosphate alarmone. The RSH/Rel enzymes typically utilize GDP and GTP substrates, with those from Gram-positive species displaying a higher affinity for GTP (34). Characterized RSH/Rel enzymes have little to no affinity for GMP *in vitro* (34, 35, 47). RelA from E. coli has been shown to be allosterically activated by pppGpp, ppGpp, and pGpp (46, 60 to 62).

Alarmones are cleared from the cytoplasm via removal of the 3′ pyrophosphate largely through hydrolysis by the hydrolase domains of bifunctional RSH/Rel enzymes (35, 58, 63). Characterized RSH/Rel hydrolases are capable of dephosphorylating the 3′ position of pppGpp, ppGpp, or pGpp, even though they do not generate the triphosphate alarmone (39, 46) Alarmone hydrolysis is crucial, as without it, cessation of cell growth and replication is irreversible. In many strains, individual alarmone synthetase genes may be deleted or mutated, or the entire suite of alarmone metabolism genes may be deleted to create alarmone-null strains. Such mutants exhibit metabolic dysregulation, impaired virulence, and stress survival and are frequently attenuated for virulence in animal infection models (64) However, alarmone hydrolase genes are frequently essential if any functional synthetase genes are intact in the genome (65 to 67).

### Small alarmone synthetases.

In addition to a single RSH enzyme, many Gram-positive Firmicutes (also known as Bacillota) also encode one or two monofunctional small alarmone synthetase (SAS) enzymes with high homology to the synthetase domain of RSH/Rel enzymes (13, 68). There are two major families of SAS, RelQ (also known SAS1, SasB, or YjbM), and RelP (alternately, SAS2, SasA, or YwaC) (2, 10, 13, 14). RelQ homologs from Bacillus subtilis and Enterococcus faecalis are allosterically activated by pppGpp (46, 59, 69). The majority of Firmicutes encode both RelQ and RelP, although some encode only RelQ (10). The RelZ protein from Mycobacterium smegmatis contains an SAS domain and an RNase H domain and is the first identified SAS with an additional domain whose activity is affected by alarmone synthesis (39, 70). SAS generally utilize both GDP and GTP with various affinities when the activity of purified protein is assessed *in vitro* by autoradiography, high performance liquid chromatography (HPLC), or ion-exchange chromatography, and SAS enzymes from E. faecalis, Cornyebacterium glutamicum, and B. subtilis can additionally use GMP as a substrate to synthesize a triphosphate alarmone (pGpp) (13, 33, 37, 39, 40, 45 to 47). RelZ is currently the only SAS shown to process GMP with higher affinity than GDP or GTP (39).

Organisms that encode SAS enzymes appear to exhibit bimodal (p)ppGpp metabolism, with RSH/Rel enzymes synthesizing or degrading (p)ppGpp very rapidly in response to conditional signals, and SAS enzymes exhibiting slower, constitutive synthesis (2, 51). In B. subtilis and E. faecalis, RSH activity results in dramatic changes to (p)ppGpp levels to induce or halt the stringent response. In contrast, SAS enzymes do not appear to induce a stringent response in the absence of RSH activity, but rather produce smaller fluctuations in (p)ppGpp levels in order to regulate different processes such as guanosine metabolism and to influence the kinetics of RSH-dependent alarmone accumulation (1, 2, 7, 71).

### Small alarmone hydrolases.

Because alarmone synthetases are toxic in the absence of alarmone hydrolases, the discovery of monofunctional SAS in addition to the bifunctional RSH-family enzymes strongly suggested the possibility that monofunctional small alarmone hydrolases (SAH) could also exist (10, 72). The first confirmed bacterial SAH, RelH_Cg_ from C. glutamicum, was recently characterized and shown to hydrolyze the 3′ pyrophosphates of all three guanosine alarmones *in vivo*, although deletion of the *relH_Cg_* gene had minimal effects on cell growth and the gene has not been highly conserved during the evolution of the genus *Corynebacterium* (38). RelH_Cg_ is most active against ppGpp, while the RSH homolog from this organism, Rel_Cg_, exhibits the most potent hydrolase activity against pGpp, suggesting that SAH and RSH enzymes could modulate the ratios of the different alarmones through their differential substrate affinities, just as SAS and RSH enzymes do (38). Both enzymes are inhibited by high-substrate concentrations, which may be necessary to avoid a futile synthesis-hydrolysis cycle and allow rapid alarmone accumulation to induce and sustain a stringent response (38). Substrate inhibition of the hydrolase domains could stabilize the stringent response until hydrolysis is activated by still-uncharacterized regulatory interactions.

### Alarmone-modifying hydrolases.

Some hydrolases modify alarmones without abolishing their two-site phosphorylation. The guanosine pentaphosphate phosphohydrolase GppA converts pppGpp to ppGpp and is thought to balance the relative levels of the two alarmones when the ratio of available GTP and GDP substrates is unsuited to the needs of the cell, or when the effectors expressed by a given species are more responsive to either the pentaphosphate or tetraphosphate alarmone (Fig. 1) (29, 48 to 50). The Nudix enzyme family hydrolyzes a diverse set of substrates that each contain a nucleotide diphosphate linked to some other moiety X, and some of them have been shown to target alarmones (73). Nudix hydrolases from Thermus thermophilus and E. coli degrade both the 3′ and 5′ pyrophosphates of ppGpp, generating pGpp and ppGp intermediates before producing pGp (74, 75). This activity is thought to remove alarmones from bacterial cytoplasm. Deletion of the T. thermophilus *ndx8* hydrolase results in ppGpp accumulation during exponential growth (74). Overexpression of the E. coli Nudix hydrolase genes *nudG* or *mutT* can complement deletion of the *spoT* hydrolase gene to relieve alarmone-mediated growth suppression (76). The E. coli Nudix proteins appear to deplete cytoplasmic pppGpp more aggressively than ppGpp. In contrast, the Nudix-family hydrolase NahA from B. subtilis specifically converts (p)ppGpp to pGpp with no apparent activity toward the 3′-pyrophosphate (Fig. 1) (33, 43, 77). Like NudG and MutT, NahA seems to demonstrate a substrate preference for pppGpp, as NahA appears to selectively convert pppGpp produced by the SasA/RelP synthetase but not ppGpp produced by the SasB/RelQ synthetase (77). pGpp accumulation is strongly decreased in a strain lacking *nahA*, indicating that NahA hydrolase activity rather than direct synthesis is the predominant source of the triphosphate alarmone (43).

### Effectors.

Alarmone-sensing effectors vary among species but include DNA primase, transcriptional regulators, GTPases involved in ribosome maturation, and many kinases that govern purine biosynthesis pathways (20). DRaCALA screens of open reading frame libraries and affinity-capture assays performed on whole-cell lysates have identified a number of alarmone-binding proteins, many involved in the regulation of purine biosynthesis and ribosome assembly (31 to 33, 75, 78). Some of these assays were limited to ppGpp or 1:1 mixes of ppGpp and pppGpp, while some included pGpp (Table 1). Many effectors were identified that bound two or three nucleotides, some with comparable affinities and some with a marked preference for one species. Notably, seven *Staphyloccocus aureus* proteins each exhibited slightly higher affinities for pppGpp than for ppGpp in whole-cell lysates, but four of them exhibited much higher affinities for ppGpp when purified, indicating that apparent binding affinities can be influenced by environmental conditions (32).

**TABLE 1:** Summary of recent experiments screening for alarmone-binding effector proteins^*a*^

Alarmone	Corrigan et al., 2016 (32)	Zhang et al., 2018 (75)^*b*^	Wang et al., 2019 (78)	Yang et al., 2020 (33)	Haas et al., 2022 (31)	
Method	DRaCALA	DRaCALA	Affinity capture	DRaCALA	Affinity capture	
Species	S. aureus	E. coli	E. coli	B. anthracis	E. coli	S. typherium
pGpp	n.t.	n.t.	n.t.	0	5	n.t.
ppGpp	0	n.t.	56	3	33	37
pppGpp	0	n.t.	n.t.	2	21	n.t.
pGpp +ppGpp	n.t.	n.t.	n.t.	3	10	n.t.
pGpp +pppGpp	n.t.	n.t.	n.t.	0	1	n.t
ppGpp +pppGpp	7	20	n.t.	12	24	n.t
all	n.t.	n.t.	n.t.	13	13	n.t

a Diffusion-based screening for the ability to bind radiolabeled alarmones and pulldown assays using immobilized alarmones have both been used to screen cell lysates for alarmone-binding proteins. The number of proteins that bound or were pulled down from bacterial cytoplasm by each alarmone is indicated. n.t., not tested (n.t.).

b Zhang and colleagues noted that their assay did not identify 11 E. coli proteins demonstrated to bind (p)ppGpp in previous works but did identify 12 previously unknown proteins.

**FIG 2:**
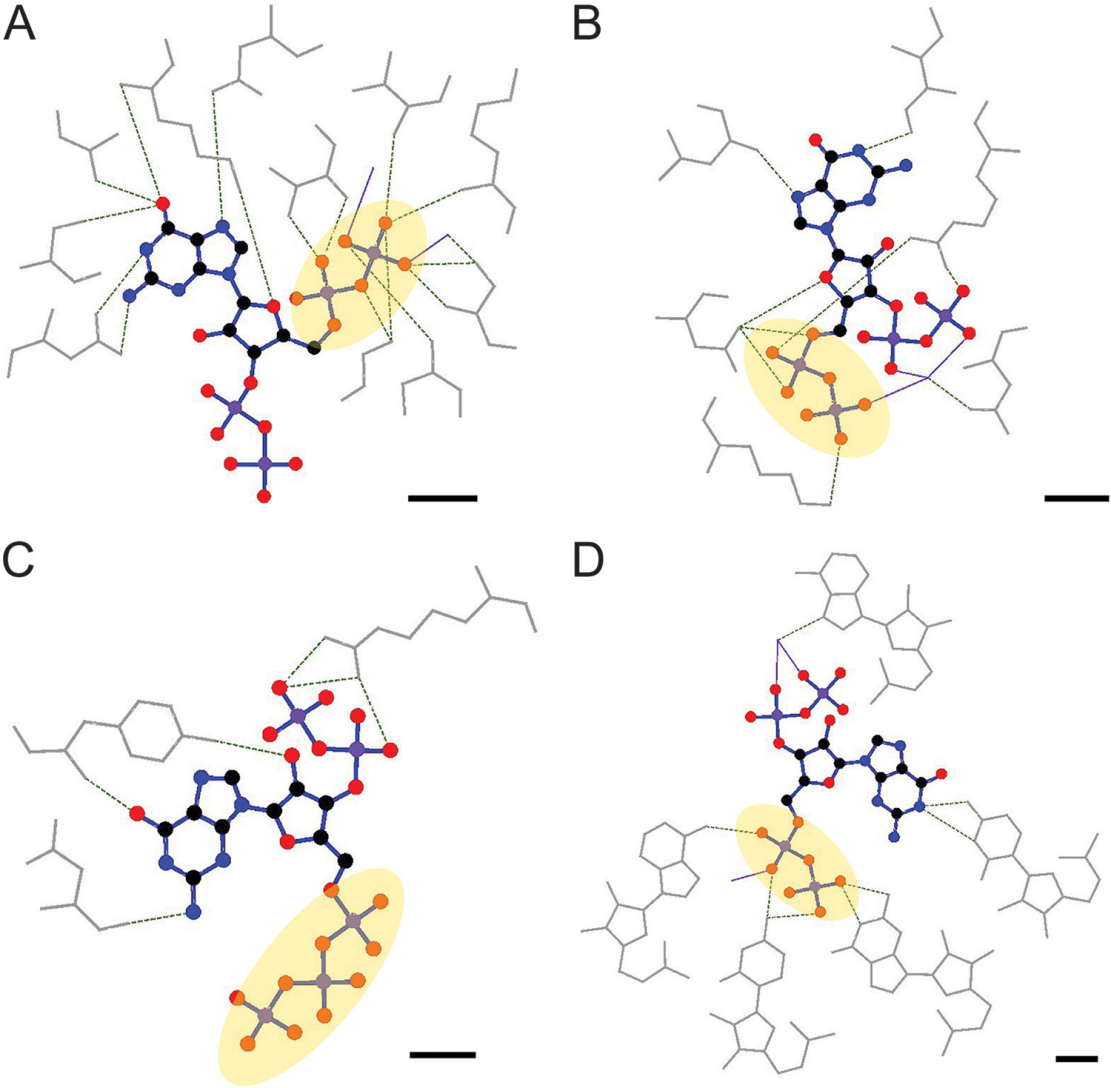
Alarmones complexed with effectors show great diversity in their 5′ phosphate groups. Alarmones and the residues they contact within their protein or RNA binding pockets are pictured. Protein and RNA residues are shown in gray. Hydrogen bonds and salt-bridges are shown as dotted lines. (A) Crystal structure of ppGpp bound to B. subtilis Obg (PDB: 1LNZ). The 5′-disphosphate is completely encased in a binding pocket (yellow circle) that appears sterically capable of accommodating a triphosphate, while the 3′-diphosphate is solvent exposed. This protein binds (p)ppGpp but not pGpp *in vitro* (33). The exclusion of pGpp is likely due to the direct and magnesium-mediated contacts between the protein and the 5′-β-phosphate. (B) Crystal structure of ppGpp bound to Francisella tularensis MglA-SspA (PDB: 5U51). The 5′-diphosphate contacts the surface of the protein but is solvent-exposed. (C) Crystal structure of pppGpp bound to E. coli PpnN (PDB: 6GFM). The 5′-triphosphate extends out of the binding pocket and has no protein contacts. This protein is pulled out of E. coli lysate by affinity-tagged pppGpp, ppGpp, or pGpp and does not appear to discriminate based on 5′-phosphate group size (31). (D) Crystal structure of ppGpp bound to the Sulfobacillus acidophilus ppGpp riboswitch (PDB: 6DME). The 5′-diphosphate is enveloped in a binding pocket and makes numerous direct contacts with the riboswitch. The scale bar in each image represents 3 Å. Contact maps were generated with LigPlot+.

Structures have been determined for twenty-eight unique proteins bound to ppGpp or pppGpp, including SAS and RSH synthetases, a GppA hydrolase, and effectors involved in RNA processing and polymerization, protein synthesis and modification, and purine nucleotide metabolism (5, 30, 79). No structures of protein effectors bound to pGpp have been found. Examination of the alarmone structures within these complexes reveals a high degree of conformational flexibility; when the sugar and base elements are aligned, the phosphate groups can take many conformations (30). Examination of the 5′-phosphate groups that determine alarmone identities within effector binding pockets reveals three types of pockets: in some complexes, the 5′-diphosphate or triphosphate is completely or almost completely encased by its receptor, which is likely to discriminate among alarmone species based on the number of phosphates at this position (Fig. 2A). In other complexes, the 5′ phosphate group is in contact with the surface of the protein in a solvent-exposed depression or trench (Fig. 2B), while in still others the 5’phosphate group has limited contact with the protein and is primarily solvent exposed (Fig. 2C).

In addition, riboswitches of the *ykkC* subtype 2a family, commonly associated with genes for amino acid biosynthesis, bind alarmones (80). A riboswitch found in the 5′ untranslated region of the Thermosediminibacter oceani *ilvE* gene binds ppGpp and pppGpp with equal micromolar affinity *in vitro*, and ppGpp binding by the *ilvE* riboswitch from Desulfitobacterium hafniense regulates *ilvE* transcription termination (80). The affinities of the *T. oceani* riboswitch for GMP, GDP, and GTP are 4,000-fold, 2,000-fold, and 5,000-fold lower than that for (p)ppGpp, highlighting the importance of the 3′ diphophate group for riboswitch ligand recognition. The same riboswitch binds pGp with 2,000-fold lower affinity than ppGpp, showing that the distinction between mono- and diphosphate groups at both positions is significant (80). Crystal structures of a ppGpp-binding riboswitch from Sulfobacillus acidophilus reveal that both diphosphate groups contact the riboswitch, and beta-phosphate of the 5′-group participates in multiple hydrogen bonds, suggesting that the riboswitch could accommodate a 5′-triphosphate but would be likely to discriminate against a 5′-monophosphate (Fig. 2D) (81). The ability of 30 predicted alarmone-binding riboswitches to bind ppGpp or pppGpp in a modified DRaCALA assay was recently assessed, and 25 showed binding activity *in vitro* (53). Seventeen of these riboswitches bound ppGpp and pppGpp with similar (less than 2-fold difference) affinities, while seven had markedly higher affinity for ppGpp and one had higher affinity for pppGpp (53). Analysis of structural models of these riboswitches revealed several elements that appear to correlate with the ability to bind pppGpp; a conserved ACA motif at the end of the P1 stem-loop and the length of the P1 stem-loop are both speculated to affect conformational flexibility of the binding pocket for the 5′-phosphate group, thus its ability to physically accommodate a triphosphate (53). As with protein effectors, no binding studies have been performed with pGpp and ribonucleotide effectors, so the degree of functional independence or redundancy of the triphosphate alarmone remains to be experimentally defined.

## AN EVOLVING UNDERSTANDING OF THE TRIPHOSPHATE ALARMONE pGpp

### Many paths to pGpp.

Until recently, pGpp synthesis had only ever been detected concurrently with (p)ppGpp synthesis. RelZ from *M. smegatis* is currently the only synthetase enzyme known to have a higher affinity for GMP than GDP or GTP, but it is still capable of ppGpp synthesis, and the cellular alarmone pool is dominated by pppGpp produced by the M. smegmatis RSH/Rel enzyme (39). The primary source of pGpp in the B. subtilis alarmone pool is postsynthetic processing of (p)ppGpp by NahA rather than direct synthesis from GDP/GTP (33, 43, 77). pGpp is typically present in the cytoplasm at much lower levels than (p)ppGpp (43). However, Clostridioides difficile encodes an RSH homolog and a single RelQ SAS, and both enzymes exclusively produce pGpp and are incapable of generating longer alarmones *in vitro* (40). The clostridial enzymes generate the triphosphate alarmone despite the fact that RSH_Cd_ utilizes GDP as its sole substrate and RelQ_Cd_ utilizes GDP and GTP, with neither enzyme capable of synthesizing alarmones from GMP precursors (40). This mismatch between the 5’phosphate groups of substrates and products is due to a previously undocumented variation on canonical alarmone synthesis. The clostridial synthetases, when incubated *in vitro* with ATP, GDP/GTP, and no other enzymes, both hydrolyze the 5′β-phosphate bond of their guanosine substrates as a necessary step for pyrophosphotransfer to the 3’hydroxyl group (40). The clostridial enzymes cannot utilize GMP, which has no β-phosphate, or GDPβS, a GDP analog in which the β-phosphodiester bond has been replaced with a thiophosphate bond resistant to hydrolysis. B. subtilis RelQ, which does not require guanosine 5′β-phosphate bond hydrolysis, can use both GMP and GDPβS as the substrates for alarmone synthesis (40). This requirement for a phosphate bond hydrolysis on the guanosine phosphoacceptor as well as on the ATP phosphodonor is unique among characterized alarmone synthetase enzymes. ^31^P nuclear magnetic resonance (NMR) studies reveal that neither RSH_Cd_ nor RelQ_Cd_ hydrolyzes guanosine β-phosphate bonds when incubated with GTP or GDP in the absence of ATP, suggesting that either ATP hydrolysis or pyrophosphotransfer initiates modification of the guansine 5’phosphate moiety, but structural characterization of the clostridial synthetases will be necessary before the mechanism of alarmone synthesis in this organism can be defined (40).

Despite the unusual alarmone metabolism employed by C. difficile, alarmone function in this organism appears to be similar to that in other bacteria. Transcription of the *rsh* and *relQ* genes is increased by stationary-phase onset and extracellular stress, and reduction of RSH_Cd_ translation by RNA interference or of RSH catalysis by the competitive inhibitor Relacin increases C. difficile antibiotic susceptibility (36, 40). In addition, it was recently reported that competition for nutrients during bacterial coinfection in a germ-free mouse model causes suppression of C. difficile protein translation, ribosome synthesis, and ATP production, all processes that are halted by the stringent response in other bacteria (20, 82). It is not known why this organism evolved the requirement for a second phosphate bond hydrolysis to synthesize alarmones, but it appears that pGpp can mediate conserved alarmone signaling in the absence of the longer species.

### pGpp as a distinct regulator.

While pGpp may function as the sole alarmone in C. difficile, other organisms appear to synthesize it as part of an alarmone pool dominated by ppGpp and/or pppGpp. It is difficult to study an individual component in a pool of partially redundant signals, and pGpp was often overlooked in studies designed to probe the extent of independence or functional redundancy between ppGpp and pppGpp. Alarmone-binding effectors were originally identified by probing E. coli open reading frame libraries with radiolabeled ppGpp and pppGpp in differential radial capillary action of ligand assays (DRaCALAs) or by pulling interacting proteins out of E. coli cell lysates with affinity-labeled ppGpp (Table 1) (75, 78). These assays identified multiple target proteins involved in alarmone metabolism as well as ribosome synthesis and function and purine nucleotide homeostasis (75, 78). More recently, pulldown assays of E. coli lysate with affinity-conjugated pGpp, ppGpp, and pppGpp identified forty-seven potential effectors that bind two or three forms of alarmone and fifty-nine that appear to select a single alarmone based on the number of 5′-phosphates (Table 1) (31). Of the twenty-nine proteins pulled down by pGpp, 13 bind all alarmones, 10 bind pGpp and ppGpp, one binds pGpp and pppGpp, and five bind pGpp exclusively, including a transcriptional regulator previously found to be upregulated by oxidative stress (31).

The distinct biological role of pGpp within a mixed pool of guanosine alarmones has been best defined in *Enterococcus* and *Bacillus* species, which encode three synthetases and metabolize all three forms of guanosine alarmone (44, 46, 83). RSH/RelA has a high affinity for GTP and mainly contributes pppGpp to the cellular alarmone pool when activated. In *Bacillus* species, SasA/RelP preferentially utilizes GTP to generate alarmones that are modified to pGpp by the NahA hydrolase, while SasB/RelQ is allosterically activated by pppGpp but preferentially uses GDP to synthesize ppGpp (33, 43, 46, 59, 77). During the transition to stationary phase when nutrients become limited, roughly half of wild-type cells display little to no protein synthesis activity. Deletion of B. subtilis *sasA/relP* increases the number of these translationally dormant cells in the population. Deletion of *sasB/relQ* reduces translational dormancy, suggesting that the ppGpp produced by SasB/RelQ negatively regulates protein synthesis in a subset of cells, generating metabolic heterogeneity in a genetically identical bacterial population. Population heterogeneity is reduced as strongly by mutation of the SasB/RelQ allosteric pppGpp binding site as by deletion of the gene, suggesting that pppGpp generated by RSH/RelA influences translational regulation via ppGpp produced by SasB/RelQ (77). pGpp does not directly affect SasB/RelQ activity *in vitro*, but it does prevent pppGpp from stimulating it, establishing a model in which pppGpp and pGpp are opposing regulators whose net activity determines how much ppGpp is produced (77). This model is not complete, as it does not explicitly account for conversion of the pppGpp produced by SasA/RelP into pGpp *in vivo*; NahA is the only hydrolase known to modify alarmones in B. subtilis, but deletion of *nahA* does not affect population heterogeneity, suggesting that enzymes relevant to this regulatory pathway have yet to be determined (33, 77). Low levels of pGpp can be detected in the cytoplasm of a *nahA-*deficient B. subtilis, but it is not known whether this is due to the activity of another hydrolase or to previously undetected direct synthesis of pGpp (43). Together, the three alarmones appear to allow B. subtilis to establish translationally dormant and active subpopulations. Deletion of *sasB/relQ* is presumed to reduce cytoplasmic ppGpp, resulting in a more uniformly active population, while deletion of *sasA/relP* is presumed to reduce pGpp, resulting in a more uniformly inactive population. Depletion of either regulator makes the population more homogenous, which leaves all component cells equally vulnerable to environmental perturbations (77).

While the specific effectors that allow alarmones to regulate translational heterogeneity in B. subtilis are unknown, some alarmone-binding effectors have been identified in this organism. DRaCALAs using radiolabeled pGpp as well as ppGpp and pppGpp have identified proteins that bind all three guanosine alarmones with various affinities and others that bind the canonical (p)ppGpp alarmones but exclude pGpp at physiological concentrations (33). This analysis revealed that the tetraphosphate and pentaphosphate (p)ppGpp alarmones interact with the GTPases that regulate ribosome maturation and function and with enzymes involved in purine nucleotide homeostasis, while pGpp appears to be involved in nucleotide metabolism but not ribosome assembly, allowing purine metabolism to be regulated independently of protein translation (33). In both Bacillus anthracis and E. faecalis, pGpp is a significantly more potent inhibitor of purine nucleotide synthesis than the longer alarmones (33, 46).

### Future directions: pGpp as a potential sole regulator.

The ability of pGpp to function independently of the longer, more characterized guanosine alarmones allows cells either to decouple processes regulated by the stringent response, allowing one to continue in certain circumstances while the other is inhibited, or to integrate multiple inputs, including the relative availability of GMP, GDP, and GTP, when generating a cytoplasmic pool of alarmones (33, 77). The fact that bacteria have evolved multiple pathways to generate pGpp, either by direct synthesis using GMP substrates or by modification of (p)ppGpp precursors synthesized from GDP or GTP substrates, indicates the conserved importance of the triphosphate nucleotide. C. difficile is the only organism currently known to require guanosine 5′-β-phosphate bond hydrolysis for alarmone synthesis, which necessitates exclusive synthesis of pGpp but precludes the use of GMP as a substrate (40). This suggests not only that is it beneficial for this organism to utilize pGpp rather than (p)ppGpp as an intracellular signal, but that it is additionally beneficial to abstain from generating endogenous (p)ppGpp. As alarmone metabolism has only been characterized in a limited number of organisms, it remains to be seen whether this exclusive use of pGpp is unique to C. difficile or more widespread. It does invite speculation as to what evolutionary pressures might have favored this modification of conserved alarmone metabolism.

One possibility is that the use of pGpp might allow cells growing in multispecies communities such as biofilms adhered to the intestinal mucosa to distinguish between endogenously produced alarmones and exogenous alarmones produced by nearby cells. Many bacteria respond to extracellular signals secreted by other bacterial cells to regulate multicellular processes such as biofilm formation (84). These signals may be generated by other cells of the same species in a process known as “quorum sensing.” Secreted signals can also enable cross-species communication in mixed populations (85). While alarmones are not known to be exported or perceived at the cell surface, pulldown assays with affinity-tagged alarmones have identified several putative protein effectors in the E. coli membrane fraction, including multiple confirmed or predicted transport proteins (31). The nucleotide second messenger 3′-5′-cyclic diadenylic acid (c-di-AMP) is essential for E. faecalis growth in complex media. There are no known c-di-AMP transporters, but exogenous c-di-AMP can rescue the growth defects of a c-di-AMP synthetase mutant strain in some growth media, suggesting that c-di-AMP either exported or released from lysed cells could be detected by nearby cells (86). Since alarmones accumulate in stressed cells, it seems plausible that they could be released by cell lysis if not actively exported. For C. difficile, which integrates into mammalian gut microbial ecosystems during times of antibiotic-induced dysbiosis and thrives when its neighbors are struggling, the use of pGpp could allow sensing of exogenously produced (p)ppGpp to monitor the condition of nearby bacterial cells (87).

It is also possible that guanosine alarmones produced by pathogens are recognized by host systems and that different alarmone species could be used to calibrate host immune recognition. An alarmone-null strain of Salmonella typhimurium used as a live vaccine in mice had a lower infectious dose than the wild-type strain but a significantly higher lethal dose, suggesting that while alarmones are necessary for virulence, they stimulate an immune response that is detrimental for establishing the initial infection (64, 88). The immune response of mice infected with Yersinia pestis is a mix of proinflammatory Th1-mediated and anti-inflammatory Th2-mediated activity, but infection with an alarmone-null strain of Y. pestis biases the immune response away from the proinflammatory response (89). The different responses could be caused by immune sensing of alarmones or of one of the virulence factors regulated by alarmones. Similarly, guinea pig infection with a *rel* mutant strain of Mycobacterium tuberculosis reduces the immune response compared to that stimulated by infection with wild-type M. tuberculosis (54, 90). Direct sensing of guanosine alarmones by mammalian immune systems has never been reported, but c-di-AMP and the related bacterial second messenger 3′–5′-cyclic diguanylic acid (c-di-GMP), which are not produced by mammals, are detected by the innate immune sensor protein STING, leaving open the possibility that mammalian receptors for other bacterial nucleotide signals could exist (91 to 93).

There is no experimental evidence that exogenous alarmones can be sensed by other bacteria or by mammalian immune cells. In a personal communication, Raue and Cashel have reported that E. coli is impermeable to exogenous pGpp, and to our knowledge the ability of mammalian immune or epithelial cells to sense alarmones has not been tested (29). It is also possible that the *in vitro* activity of the purified clostridial synthetase enzymes does not fully reflect activity in the cell and that these or other proteins could synthesize longer alarmones under certain conditions *in vivo.* However, the dependence of clostridial alarmone synthesis on the hydrolysis of the guanosine 3′β phosphate bond indicates that pGpp rather than (p)ppGpp plays some role in the C. difficile life cycle.

## CONCLUDING REMARKS

For a long time, guanosine alarmones were regarded exclusively as the switch that governs the bacterial stringent response, which has been referred to as a singular process because the phenotypes it governs converge across diverse clades. However, the additional roles played by basal levels of alarmones in governing growth rate and metabolism and the functional diversity exhibited by the penta-, tetra-, and triphosphate alarmones have become undeniable. It has been convenient to refer to the alarmones collectively, first as (p)ppGpp and then as (pp)pGpp when pGpp was recognized, but it is clear that pGpp signaling encompasses more than a reinforcement of the processes initiated by ppGpp and pppGpp. While much remains to be discovered about the interaction of pGpp with the longer alarmones and its independent role in diverse bacteria, it is apparent that it should be considered an individual entity rather than a nearly interchangeable part of a set.
